# Strength Parameters of Clay Brick Walls with Various Directions of Force

**DOI:** 10.3390/ma14216461

**Published:** 2021-10-28

**Authors:** Rafał Nowak, Tomasz Kania, Valery Derkach, Romuald Orłowicz, Anton Halaliuk, Ewa Ekiert, Rafał Jaworski

**Affiliations:** 1 Department of General Civil Engineering, Faculty of Civil and Environmental Engineering, West Pomeranian University of Technology in Szczecin, Piastów Ave. 50a, 70-311 Szczecin, Poland; 2 Department of General Civil Engineering, Faculty of Civil Engineering, Wrocław University of Science and Technology, Wybrzeże Wyspiańskiego 27, 50-370 Wrocław, Poland; Tomasz.Kania@pwr.edu.pl; 3 Branch Office of the “Institute BelNIIS”—Scientific-Technical Center, 224023 Brest, Belarus; v-derkatch@yandex.by (V.D.); institute@belniis.by (A.H.); 4 West Pomeranian University of Technology in Szczecin, Piastów Ave. 50a, 70-311 Szczecin, Poland; orlowicz@yandex.ru (R.O.); Rafal.Jaworski@op.pl (R.J.); 5 Department of Chemical Inorganic Technology and Environment Engineering, Faculty of Chemical Technology and Engineering, West Pomeranian University of Technology in Szczecin, Piastów Ave. 42, 71-065 Szczecin, Poland; edabrowa@zut.edu.pl

**Keywords:** clay bricks, cement lime mortar, infill masonry wall, destructive force

## Abstract

The study analyzes the anisotropy effect for ceramic masonry based on experimental tests of samples made of 25 × 12 × 6.5 cm^3^ solid brick elements with compressive strength *f_b_* = 44.1 MPa and cement mortar with compressive strength *f_m_* = 10.9 MPa. The samples were loaded in a single plane with a joint angle that varied from the horizontal plane. The load was applied in a vertical direction. The samples were loaded at angles of 90°, 67.5°, 45°, 22.5°, and 0° toward the bed joints. The most unfavourable cases were determined. It was observed that the anisotropy of the masonry significantly influences the load-bearing capacity of the walls depending on the angle of the compressive stresses trajectory. Approximation curves and equations for compressive strength, Young’s modulus, and Poisson’s coefficient were proposed. It was observed that Young’s modulus and Poisson’s ratio will also change depending on the trajectory of compressive stresses as a function of the joint angle. Experimental tests allowed to determine the failure mechanism in prepared specimens. The study allowed to estimate the masonry strength with the load acting at different angles toward the bed joints.

## 1. Introduction

Clay (or mud) has been used in the building industry since ancient times [1,2]. Clay-based building materials can be classified in many categories in terms of the preparation process and use, such as mud bricks, clay plasters, cob, and rammed earth [2,3]. Most typical clay brick structures work mainly in compression perpendicular to the bed (horizontal) joints. Therefore, their compressive strength is determined in this particular direction, according to the methodology presented in the standard [4]. It is less common for masonry to work in compression at a different angle from the joints [5,6,7,8,9,10].

An example of masonry loaded at different angles to the bed joints are walls subjected to seismic actions. The evaluation of the shear behavior of masonry walls is a fundamental step for the assessment of masonry in seismic zones [11,12]. Under lateral forces, the low tensile strength generally leads to local or global failure modes, the latter related to shear or flexural mechanisms [13]. The latest works in the field of research and modeling of masonry structures concern the influence of the value of the modulus of elasticity and Poisson ratio outside the range of 33% of the ultimate stress on the shear behavior of masonry walls. Nonlinear static analyses are commonly adopted for the evaluation of seismic performance [11,12,13,14,15,16,17]. Research on that subject has been presented by Laurenco et al. [14] with a yield criterion that includes different strengths along each material axis. The criterion includes two different fracture energies in tension and two different fracture energies in compression. This model is validated with uniform biaxial loading conditions [11,14]. Celano et al. in [15] presented research on the in-plane resistance of masonry walls by means of two modeling approaches: a finite element model and a discrete macro-element model with the use of non-linear analyses. Beconcini et al. in [12] presented a combined test procedure for the experimental characterization of masonry mechanical parameters and the assessment of the shear behavior of masonry walls.

**Figure 1:**
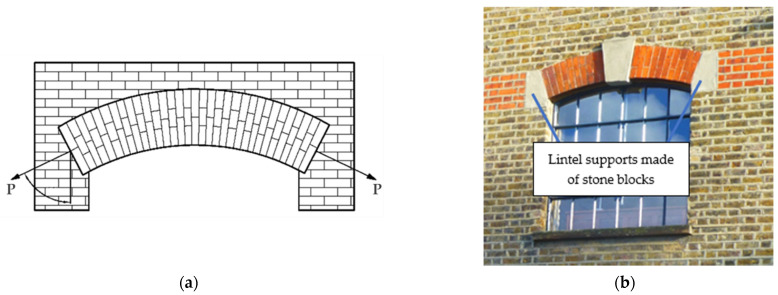
Examples of arched lintels (**a**) diagram with load (P) acting at an angle to the joints; (**b**) image of an arched lintel with stone blocks in support zones.

Another example of masonry with load (P) at an angle to the bed joints is visible in arched lintels (Figure 1a), commonly found in historic buildings. The angle of inclination of the pressure line in the support zones depends on its shape and the span-to-bow ratio. It can range from θ = 10° to 40°. As compression is applied to the wall at a lower angle of load capacity, stone blocks were sometimes required to be used as supports [18,19,20]. (Figure 1b).

**Figure 2:**
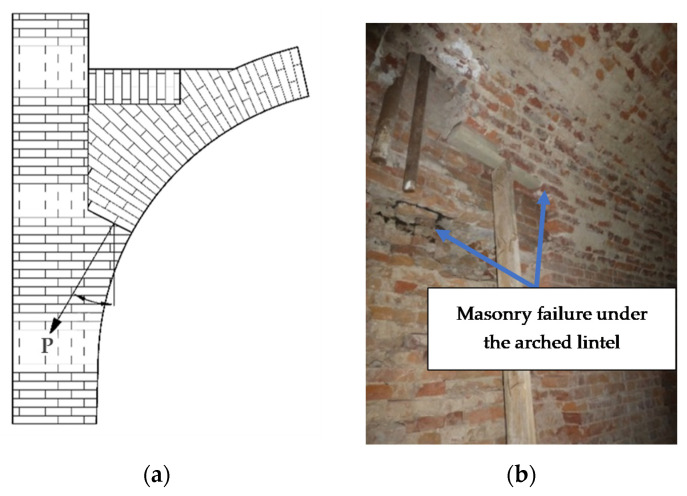
Arched lintels support zones in masonry (**a**) diagram; (**b**) example of failure mechanism.

The supports of masonry vaults also transfer the point load towards the wall at a different angle to the joints. In the case of historical buildings, vault support zones are susceptible to damage and repairs, as they transfer the most stresses (Figure 2).

In most scientific research on design procedures, only the strength perpendicular to the bed joints is usually considered. The anisotropy of the masonry is described as the ratio of the wall strength at the angle *f_c_*_,θ_ and perpendicular to the bed joints (*f_c_*_,0_). The rate depends on the material used, number of hollows, thickness, and type of the joint. The influence of masonry anisotropy is usually neglected and not analyzed. According to [21], with a more precise calibration of the calculation models, a shear test is also performed. Rarely is the strength of the masonry parallel to the bed joints tested, which may be much weaker than perpendicular [22,23,24,25,26,27,28,29,30]. Tests show that the strength parallel to the bed joints usually differs from the perpendicular strength within the limits *f_c_*_,90_/*f_c_*_,0_ = 0.2–1.2. In the study [23] obtained value was *f_c_*_,90_/*f_c_*_,0_ = 1.2, however, the models in this direction had a much lower height dimension than in the perpendicular direction—which could have influenced the results.

Even less frequently, the parameters of the wall are tested at different angles. In the studies [31,32,33,34], masonry elements were tested on a 1:2 scale for compression and tension at the following angles θ = 0°, 22.5°, 45°, 67.5°, 90° at load in both planes σ_1_ and σ_2_. For the purpose of that study, the authors used cement and lime mortar with a compressive strength of 5.55 MPa and 15.41 MPa clay bricks. The described research allowed to create calculation criteria for later different FEM (Finite Element Method) models. A different study [19] tested sand plast bricks (calcium silicate form) with a compressive strength of 23.4 MPa and 10.2 MPa cement and lime mortar with joints of approximately 5 mm. The study considered elements of the 1:2 scale in compression and tension at the same angles. The influence of the wall angle on the achieved wall strength, i.e., the degree of anisotropy, was highlighted in that study. In study [35], a failure criterion for biaxially loaded hollow blocks masonry has been researched. 1:1 scale samples of hollow clay blocks were tested, with angles as in previous studies for models with different geometries depending on the size of blocks. Similarly in study [18], but for angles θ = 0°, 15°, 30°, 45°, 60°, 75°, 90°, the tests were carried out on concrete blocks with 20% and 40% hollows, silicate blocks with 20% hollows, and clay blocks with 20% and 40% hollows. This research considered a typical cement–lime mortar. Studies allowed to estimate the degree of anisotropy of masonry for concrete and silicate blocks *f_c_*_,90_/*f_c_*_,0_ = 0.71 and for hollow clay blocks *f_c_*_,90_/*f_c_*_,0_ = 0.37.

Other structures that work in a state of compressive stress, in a different direction than indicated in the standard [4], are stiffening walls, infilling walls or elements subjected to uneven settlement of the ground. The different direction of the force action results in a complex stress state within the masonry construction, where the main axes are not parallel to the plane of the bed joints. In the case of this type of structure, its damage usually occurs as a result of exceeding its tensile strength. In residential buildings constructed in the last year in Poland, more than 95% of infilling walls were made with masonry technology [36]. Furthermore, 27.5% of the walls were made of clay elements, indicating the essence of the cracking problem that was solved in the presented research.

**Figure 3:**
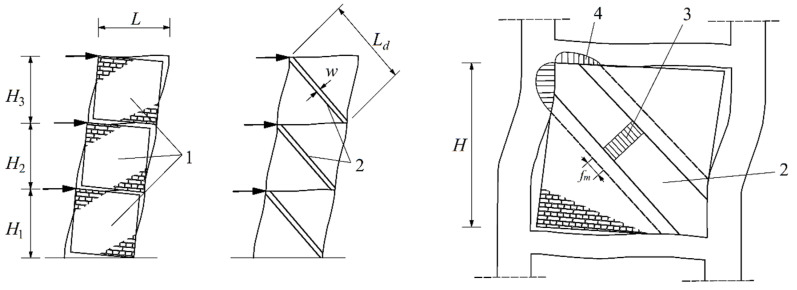
Strut model for the masonry infill wall based on [6] *L, H, H_*1*_, H_*2*_, H_*3*_*—wall geometry, 1—infill wall; 2—equivalent pinned strut; 3—stress distribution in the equivalent pinned strut; 4—contact stresses at corners; *w*—width of the strut; *L_d_*—length of the strut; *f_m_* –compressive strength of the masonry.

One of the most common calculation methods for stiffening walls in skeleton buildings is to assume a strut model. In this method, it is assumed that due to the interaction of the reinforced concrete skeleton with the walls, for the purpose of the calculation, compressed equivalent pinned strut masonry elements are being assumed. The elements with width *w* and length *L_d_* (Figure 3) play the role of stiffeners for the building [37,38]. The width of the element depends on the length of contact between the filling wall and the building skeleton [39,40,41,42,43,44,45]. Due to the masonry anisotropy discussed in the article, the actual strength of the wall will change depending on the slope of stress in relation to the plane of the bed joints of the wall. This effect will be particularly visible in walls with a low *H*/*L* ratio or in walls made of elements with vertical hollows.

In the analysis of these building elements, it is important to take into account the anisotropy of the strength parameters of the walls in relation to the direction of the compressive forces. As there are not many studies showing the mechanical properties of ceramic walls subjected to angular loads, the authors undertook this task. The novelty state of this study is the determination of the degree of anisotropy, compressive strength, the change of Young’s modulus and Poisson’s coefficient of 1:1 scale ceramic wall samples made of 25 × 12 × 6.5 cm^3^ solid bricks with compressive strength *f_b_* = 44.1 MPa.

## 2. Materials and Methods

### 2.1. Materials

The tested samples presented in this research were built using clay brick (FCP, Brest, Republic of Belarus), class 40. These are elements used for bricklaying the stiffening and infilling walls in the authors’ countries. The dimensions of the bricks are 25 × 12 × 6.5 cm^3^. For the preparation of joints, cement mortar (FCP, Brest, Republic of Belarus) with compressive strength *f_m_* = 10.9 MPa has been used. For the preparation of masonry mortars, a factory-made dry mortar mixture was used (FCP, Brest, Republic of Belarus). The thickness of the joint was about 1 cm. To determine the properties of the materials used, initial tests were conducted for bricks and mortar. Tests were performed in accordance with current standards [46,47,48]. The results are presented in Table 1.

### 2.2. Methods

**Figure 4:**
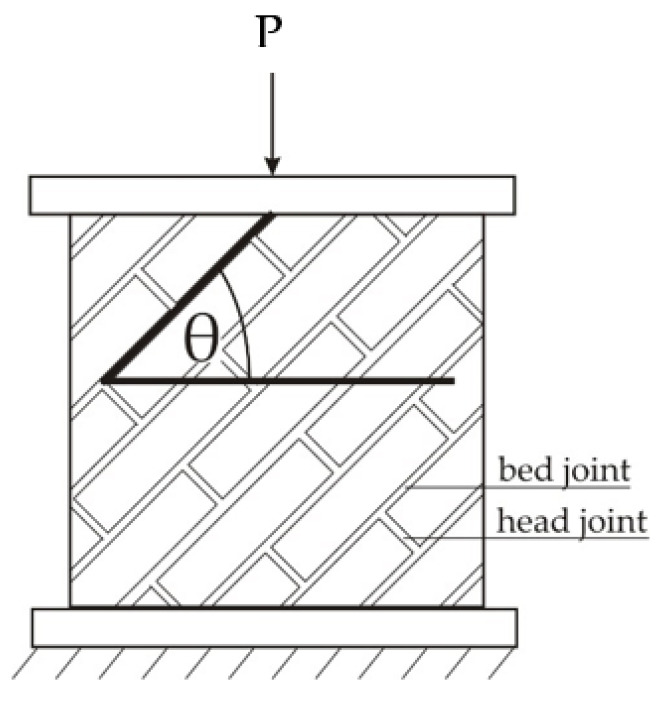
Description of θ angle measurements of tested samples.

**Figure 5:**
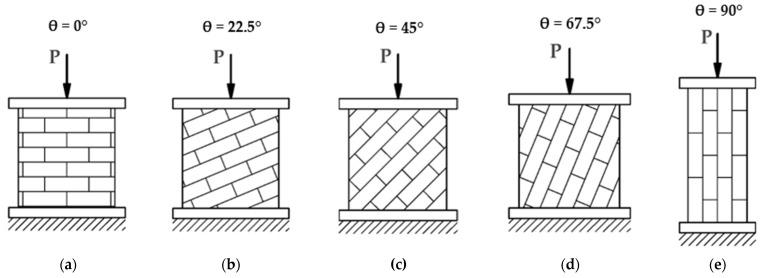
Load application diagram for compressed wall samples at different angles of bed joints (**a**) θ = 0°, (**b**) θ = 22.5°, (**c**) θ = 45°, (**d**) θ = 67.5°, (**e**) θ = 90°.

The main research program was to test 28 wall masonry panels. The specimens were made under laboratory conditions. The preparation of the panels, their curing, testing, and processing of the test results were carried out in accordance with the EN 1052-1 standard [49]. The angle of the bed joint changed: θ = 0°, 22.5°, 45°, 67.5°, 90° (Figure 4 and Figure 5). The samples had standard dimensions of 50 × 50 × 12 cm^3^, except for those with bed joints parallel to the load direction (θ = 90°) of dimensions 27 × 75 × 12 cm^3^. The dimensions of the panels were selected in accordance with the RILEM guidelines used in the other discussed works in the field of this research [35,50].

Five specimens were prepared for each joint angle, except the θ = 0°, where eight specimens were used.

The samples were built on the flat surface of the compressive strength test stand plate on a thin sand bed. Until the test, the elements were stored at a temperature of 20 °C and an air humidity <65%. The tests were carried out 28 days from the date of preparation of the samples.

**Figure 6:**
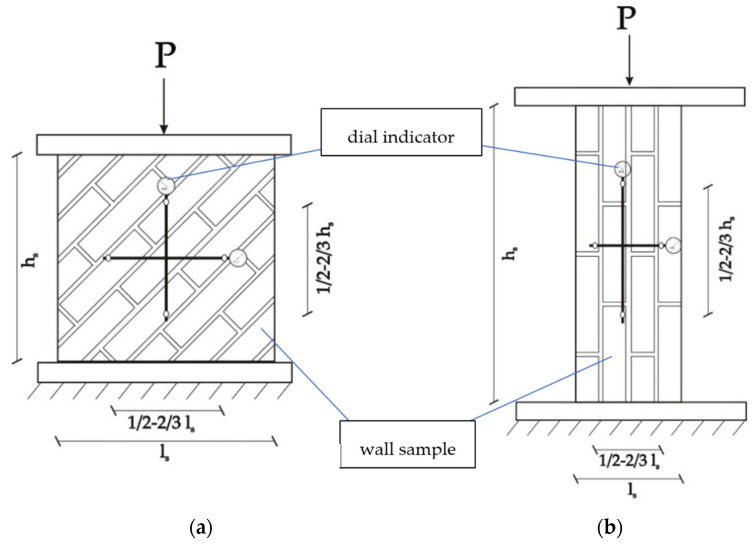
Compressive strength test with dial indicator installation, h_s_—height of the sample, l_s_—length of the sample (**a**) θ = 45°, (**b**) θ = 90°.

The models were loaded with a hydraulic actuator (Pneumat P3000, Minsk, Belarus) with a steadily increasing rate on the stand of own production, with the use of a 1250 kN hydraulic press. The samples were loaded with an increase in force equal to 12 kN per minute up to the value at which their collapse occurred. The force was measured with a dynamometer (Pneumat M, Minsk, Belarus). Dial gauges were installed on both surfaces of each sample to measure horizontal and vertical displacements (Figure 6).

Young’s modulus and Poisson’s ratio were determined in terms of the elastic work of the samples, in accordance with the requirements of the standard [49].

During the experimental tests, the destruction processes were also recorded with high-resolution cameras.

## 3. Results and Discussion

### 3.1. Results of Compressive Strength Tests

The main results obtained during the compressive strength tests are presented in Table 2.

**Figure 7:**
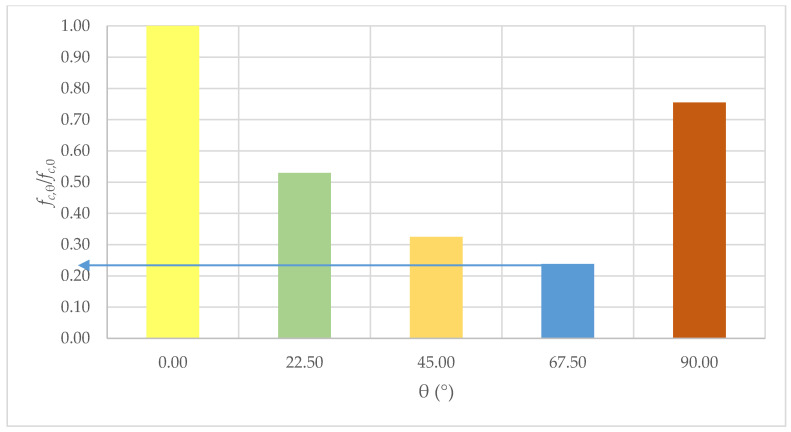
Changes in the compressive strength coefficient *f_c_*_,θ_/*f_c_*_,0_ in relation to the angle of the bed joints angle θ.

There are visible changes in the average strength of the masonry with a change in the load angle in relation to the bed joints. The highest compressive strength of 15.1 MPa was obtained for samples loaded in the direction perpendicular to the bed joints (θ = 0°). The lowest results (3.6 MPa) were obtained for the angle θ = 67.5°. The strength of the element was 4.2 times lower than the strength of the model with force acting in the direction perpendicular to the bed joints. Figure 7 presents the changes in the wall strength in relation to the reference model (θ = 0°).

As the results obtained show, minimal compressive strength should be expected for the load acting on the samples with the angle of the bed joints θ = 57.5°. Its value is limited to 21% of the compressive strength for the load acting parallel to the bed joints (θ = 0°). For the samples with angle θ = 90°, the compressive strength was limited to 75% of the strength of samples with θ = 0°.

### 3.2. Results of Deformation, Young’s Modulus, and Poisson’s Coefficient Tests

#### 3.2.1. Stress–Strain Dependencies

**Figure 8:**
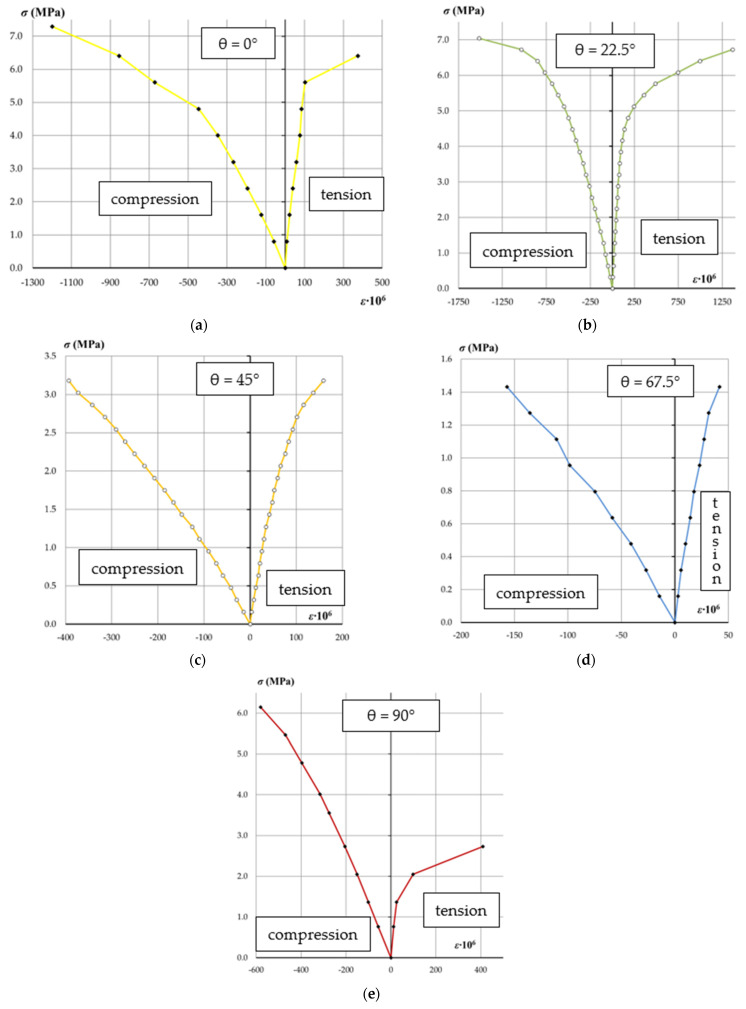
Stress–strain diagrams for compressed wall samples at different joint angles (**a**) θ = 0°, (**b**) θ = 22.5°, (**c**) θ = 45°, (**d**) θ = 67.5°, (**e**) θ = 90°.

The results of measurements of the dependence of the deformation of the tested samples in the longitudinal and transverse directions to the applied load are shown in Figure 8.

In each of the analyzed cases, the range of compressive strains of the tested samples exceeds the tensile strain values. With the increase in the value of the angle θ of the tested samples, the value of tensile strain (in the direction transverse to the direction of the force) increases. The range of compressive stresses in the area of elastic work of the wall also changes due to the different strength of the tested samples, loaded at different angles θ.

#### 3.2.2. Young Modulus Measurements

The results obtained during the Young’s modulus measurements are presented in Table 3.

**Figure 9:**
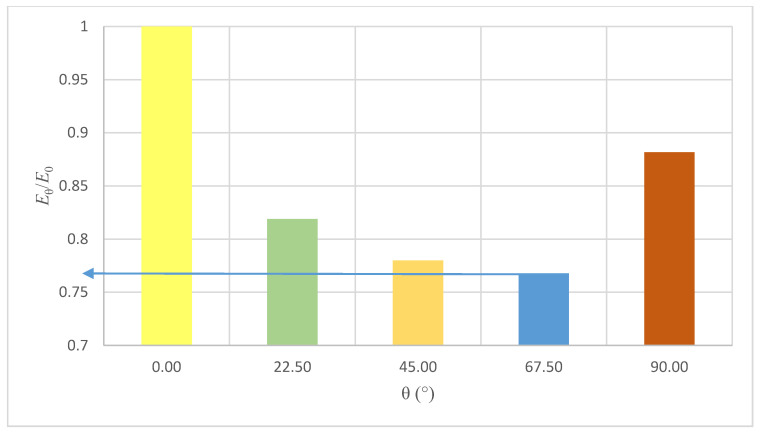
Changes in Young’s modulus coefficient *E*_θ_/*E*_0_ in regard to the angle of the head joints angle θ.

The highest value of the modulus of elasticity (*E* = 11.146 GPa) was obtained for samples loaded perpendicularly to horizontal joints (θ = 0°). The lowest value of *E* = 8.563 GPa was obtained for the samples with angle θ = 67.5°. Figure 9 presents the changes in the Young’s modulus of the wall in relation to the reference model (samples θ = 0°) in dependence of the bed joints angle θ.

The lowest value of the coefficient *E*_θ_/*E*_0_ has been obtained for samples with joints rotated at an angle θ = 67.5°.

#### 3.2.3. Measurements of the Poisson’s ratio

The results of the Poisson’s coefficient measurements are presented in Table 4.

**Figure 10:**
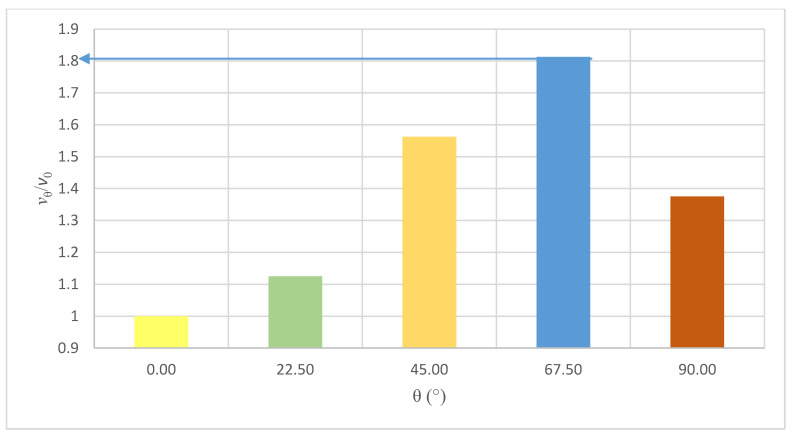
Changes in Poisson’s ratio *ν*_θ_/*ν*_0_ in regard to the angle of the bed joints θ.

The lowest value of the Poisson coefficient *ν_xy_* = 0.156 was obtained for samples with a load acting perpendicularly to the bed joints. The highest value *ν_xy_* = 0.290 was acquired with the samples rotated with angle θ = 22.5°. Figure 10 presents the graph with an approximate dependence between coefficient *ν*_θ_/*ν*_0_ and the load acting on samples with bed joints rotated with angle θ.

**Figure 11:**
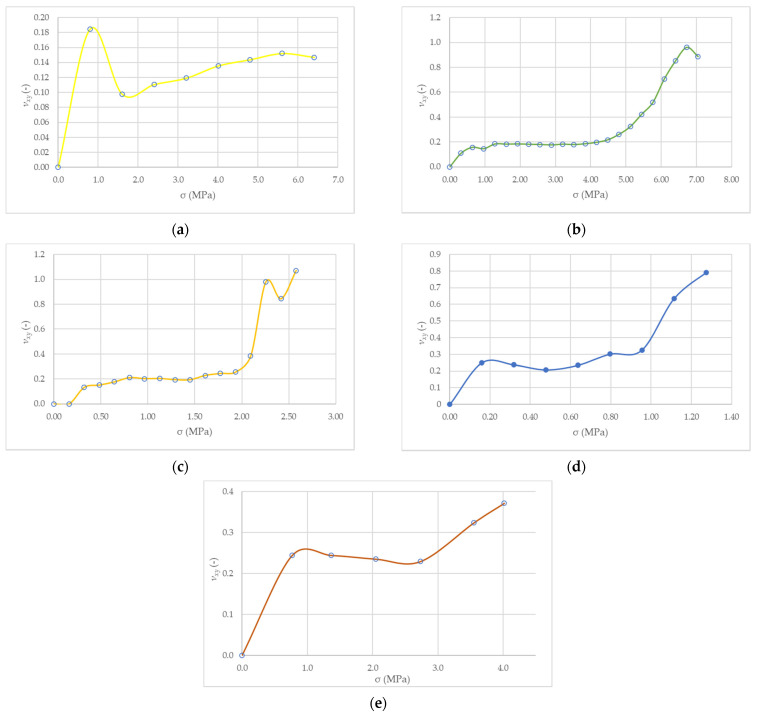
Representative Poisson’s ratio–stress curves of tested wall panels at different joint angles (**a**) θ = 0°, (**b**) θ = 22.5°, (**c**) θ = 45°, (**d**) θ = 67.5°, (**e**) θ = 90°.

The maximum value has been obtained with angle θ = 67.5°, with coefficient *ν*_θ_/*ν*_0_ = 1.81. Figure 11 presents the representative Poisson’s ratio–stress curves of tested wall panels at different angles θ.

The difference in the course of the curves is visible both in the value of the Poisson number after their stabilization from the initial stresses and in the range of their subsequent increases.

For the angle θ = 0°, the course of the curve (after stabilization in the range of the initial stress increase) is characterized by a slight upward trend from *ν_xy_* = 0.10 to 0.15 at the end of the measuring range reaching 40–50% of the limit stress. Samples with bed joints turned by the angle θ = 22.5° are characterized by a parallel course of the dependence *ν_xy_*-*σ* in the range up to 40–60% of the maximum stresses. Then the value of the Poisson number increases with the increase in deformation of the samples in the horizontal direction and the formation of vertical cracks. Along with increasing the value of the angle θ to a value of 67.5°, the value of the Poisson’s ratio increases. For the angle of θ = 45°, it reaches the value of 0.2 with a load equal to 15% of the limit value and 0.25 with 30% of the maximum stress. This increase is related to the formation of the first cracks. As stress increases further, the Poisson ratio also increases. Strain values in the horizontal direction become equal to the vertical direction at stresses equal to half of the limit values. In the case of the tested panels θ = 67.5°, the initial value of the Poisson’s ratio is characterized by the highest value among all the tested wall models (*ν_xy_* = 0.29) up to the value of 30% of the ultimate stresses. Above 30% of stresses, the value of *ν_xy_* increases. In the tested range of deformations, the Poisson’s number reaches a value of 0.8 at stresses equal to 40% of the limit value. For θ = 90°, in the range from 5 to 30% of the ultimate stress, the value of *ν_xy_* was within the range of 0.22 to 0.25. Above the value of 25–30% of the limit stress, the Poisson number increases, reaching a value of 0.37 at 40% of the limit stress.

### 3.3. Failure Mechanism

**Figure 12:**
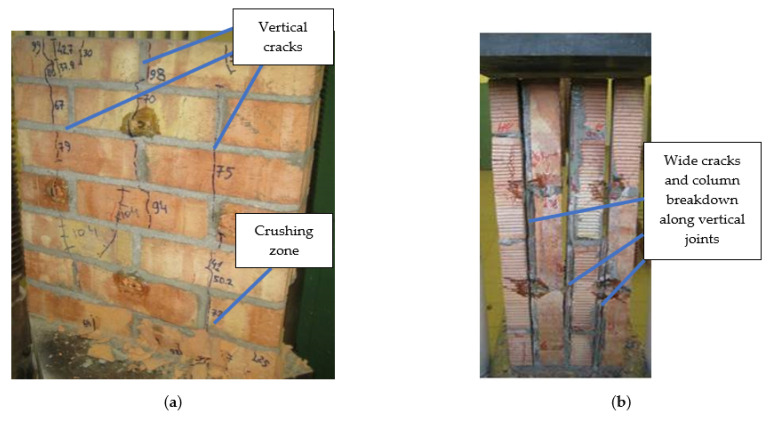
Failure mechanism of wall specimens θ = 0° (**a**) and θ = 90° (**b**).

The failure mechanism of the specimens depended on the angle of the bed joints θ. In the case of samples with a load acting in the perpendicular direction to the bed joints (θ = 0°), the failure was caused by vertical cracks. The first cracks appeared in the first and last rows of clay elements. At a load varying from 0.7 to 0.8 of the observed strength of the specimens, cracks appeared throughout the height of the specimens. After reaching the maximum stress, the width of the crack increased and local crush zones formed in the lower part of the samples (Figure 12a).

For the angle θ = 90° (Figure 12b), the adhesion of the mortar and the brick was decisive. The failure occurred by breaking the contact zone between the brick and the mortar, which caused a loss of stability of the element. The first cracks were formed with a load of 10 to 20% of the wall strength. The initial crack length was 100–150 mm and its opening was 0.1 to 0.15 mm. With a load value of 40 to 60% of the breaking limit, the cracks passed through the entire height of the samples, dividing their surface into four columns. After reaching maximum load, there was a sharp increase in the width of all previously formed cracks. The collapse was caused by the loss of stability of the individual columns.

**Figure 13:**
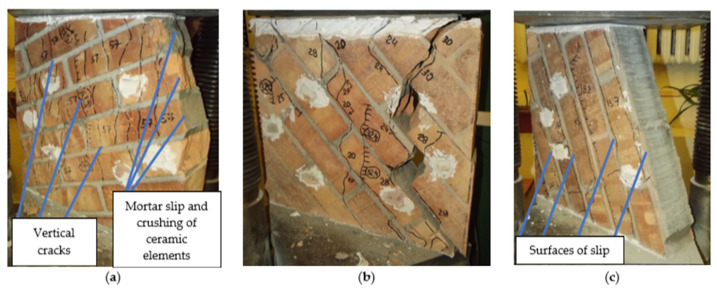
Mechanism of failure of wall specimens θ = 22.5° (**a**), θ = 45° (**b**), and θ = 67.5° (**c**).

The failure mechanism of the samples loaded at an angle of θ = 22.5° (Figure 13a) was mixed. The main failure is caused by vertical cracks that pass through the bricks and the joins. The first cracks formed at stresses of 40 to 60% of the maximum stress values. After reaching the maximum stresses, the cracks passed through the contact zone of the clay elements and the mortar, and through the brick section. The collapse was accompanied by an increase in the width of the cracks and by crushing fragments of the samples.

For angle θ = 45° (Figure 13b), mortar slip becomes a decisive cause of damage. The first cracks were formed under a load value from 25 to 35% of the observed strength of the wall samples. The initial length of the cracks did not exceed half of the height of the specimen. The width of the crack was 0.15 to 0.20 mm. With the increase in force to 80% of the tested strength, new cracks occurred, passing through the joints and the clay elements of the samples. After reaching maximum stress, the specimens collapsed as a result of cracks that ran through the entire height of the elements and chipping of the wall fragments.

The failure mechanism of the samples loaded at an angle θ = 67.5° (Figure 13c) mainly on the slip of the mortar in the adhesion plane with the clay elements. The first cracks appeared at loads ranging from 0.3 to 0.5 of the ultimate force. The length of the cracks ranged from 70 to 90% of the height of the specimen, and their width ranged from 0.15 to 0.20 mm. The cracks ran through both the supporting joints and the head joints. After reaching the maximum stress, all samples were damaged due to the sliding of the elements at the point of contact between the mortar and bricks, as shown in Figure 13c.

### 3.4. General Discussion of the Results

**Figure 14:**
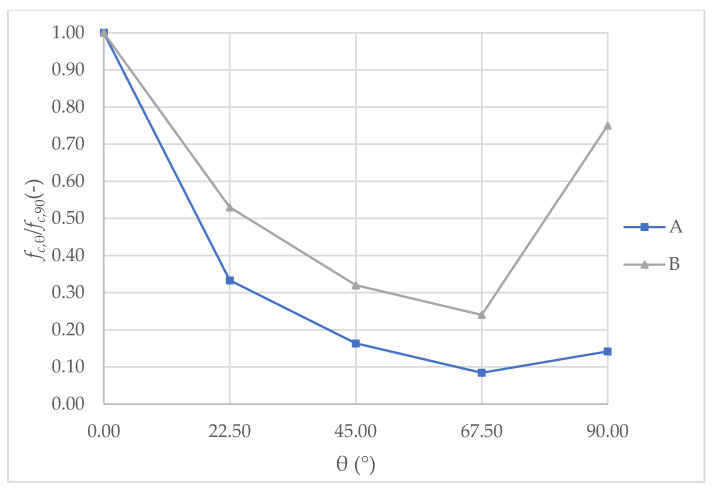
Changes in relative compressive strength *f_c_*_,θ_/*f_c_*_,0_ in regard to angle θ of A— hollow clay blocks in biaxial compression based on [35], B—walls of tested clay bricks.

The results obtained were compared with the earlier studies on brick walls quoted in the review of the literature. The studies presented in the articles [18,31,32,33,34] do not provide results of load capacity that could be used as a comparison. Previous research of different types of clay bricks, hollowed, biaxially loaded walls [35] has shown different dependencies of strength anisotropy. This comparison is presented in Figure 14.

In the range of θ = 0° to 67.5°, the course of the dependency curve for both types of ceramic walls shows some similarities. Coefficient *f_c_*_,67.5_/*f_c_*_,0_ (for θ = 67.5°) reached a value of 8% for the biaxially compressed, hollowed blocks walls, and 24% for those tested in this research, uniaxially compressed brick walls. The load-bearing capacity for θ = 90° of the walls of clay blocks with hollows, in biaxial compression state of compression (*f_c_*_,90_/*f_c_*_,0_ = 14%) [35] is much lower than the research for uniaxially loaded brick walls (*f_c_*_,90_/*f_c_*_,0_ = 75%).

A comparison made between this study and studies [19,35] indicated that the most unfavorable loading angle is between θ = 45°–67.5°. The decrease in stiffness is also the highest in this direction, based on the research presented in this study.

The designed load-bearing capacity of masonry walls loaded at an angle to the bed joints should be significantly reduced. For estimating the strength of brick walls with differentiated load orientation, presented test results can be used. In the tests presented in this article, the reduction in strength in extreme cases of rotation was up to 24% of the initial value.

FEM structure modeling is often used for structural analysis of historic masonry buildings. The detailed micro-modeling or simplified micro-modeling is time consuming and requires a lot of calibration data from the existing structure. Very often, such detailed data are difficult or impossible to collect. To simplify these problems, masonry can be calculated as a homogenous composite with macro-modeling technics [11]. Experimental data provided by us could help to implement the change of stiffness of the model and the change of load-bearing capacity in the case of stresses trajectory direction towards the bed joints.

Future research should focus on the analysis of the degree of anisotropy of the masonry of the different types of wall elements. It is important to continue these types of analyses for a wider group of materials. Analyses that allow for the approximation of mechanical properties of the walls loaded in different directions in their surface plane allow, for example, for the proper calculation of the strength of the infilling walls interacting with building structures.

## 4. Conclusions

The study determined the degree of anisotropy for masonry samples made of solid bricks and cement mortar. The models were made on a 1:1 scale. Based on the research presented, the following conclusions were drawn.

- The mechanical properties of the clay brick walls significantly depend on the angle of the compressive force.
- On the basis of the performed tests, the dependencies allowed for the calculation of the mechanical properties for the designed brick walls operating in a uniaxial state of compressive loads were determined.
- The results obtained, together with the results of other studies, allowed a better understand of the problem regarding the degree of anisotropy in the masonry. The results allowed assessing the significance of strength loss for cases with the load applied at an angle to the bed joints.
- A minimal compressive strength has been obtained for the load acting at an angle θ = 67.5°. Its value is limited to 24% of the compressive strength for the load acting parallel to the bed joints (θ = 0°).
- The lowest modulus of elasticity was researched on the walls with the bed joint angle of θ = 67.5°. Its value was evaluated as 77% of the initial value (measured for samples θ = 0°).
- The maximum of the Poisson’s ratio was obtained with angle θ = 67.5°, with coefficient *ν_θ_*/*ν_0_* = 181%.
- The conducted tests allowed identifying and describing the failure mechanism of the tested wall panels.

## Figures and Tables

**Table 1:** Initial material test results.

No.	Load Diagram	Test	Material	Result
A	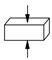	Compressive strength (EN 772-1 [46])	Brick	*f_b_* = 44.1 MPa
B	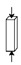	Young and Poisson	Brick	*E* = 11,850 MPa ν = 0.11
C	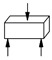	Flexural strength	Brick	*f* = 3.2 MPa
D	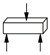	Flexural strength (EN 1015-11 [47])	Mortar	*f* = 3.3 MPa
E	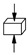	Compressive strength (EN 1015-11 [47])	Mortar	*f_m_* = 10.9 MPa
F	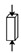	Young and Poisson	Mortar	*E* = 10,580 MPa ν = 0.17
G	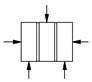	Shear strength (EN 1052-3 [48])	Masonry	*f_v_*_o_ = 0.50 MPa tg(α) = 0.5

**Table 2:** Compressive strength testing results of wall samples with different angles of bed joints θ.

Load Angle	Ordinal No	Observed Compressive Strength	Mean Compressive Strength	
		*f_c,_*_obs_ (MPa)	*f_c_*_, mean_ (MPa)	Coefficient of variation CoV (%)
θ = 0°	A1	16.79	15.1	9.0
	A2	16.94		
	A3	12.16		
	A4	13.98		
	A5	16.14		
	A6	13.18		
	A7	16.39		
	A8	15.08		
θ = 22.5°	B1	6.69	8.0	11.5
	B2	7.78		
	B3	9.27		
	B4	8.12		
	B5	7.99		
θ = 45°	C1	5.72	4.9	13.0
	C2	3.95		
	C3	4.96		
	C4	5.16		
	C5	4.83		
θ = 67.5°	D1	3.78	3.6	18.1
	D2	2.58		
	D3	3.42		
	D4	4.08		
	D5	4.22		
θ = 90°	E1	12.44	11.4	7.4
	E2	10.89		
	E3	10.31		
	E4	11.96		
	E5	11.40		

**Table 3:** Young’s modulus test results of wall samples with different angle of bed joints θ.

Load Angle	Ordinal No.	Observed Young’s Modulus in Compression	Mean Value of The Young’s Modulus	
		*E*_*y,*obs_ (MPa)	*E*_*y,*mean_ (MPa)	CoV (%)
θ = 0°	A1	9058	11,146	17.81
	A2	8800		
	A3	8750		
	A4	12,605		
	A5	13,330		
	A6	12,719		
	A7	12,759		
	A8	11,144		
θ = 22.5°	B1	8327	9127	15.16
	B2	7982		
	B3	10,954		
	B4	10,272		
	B5	8099		
θ = 45°	C1	7393	8696	11.16
	C2	9240		
	C3	9454		
	C4	7921		
	C5	9470		
θ = 67.5°	D1	10,222	8563	15.77
	D2	9247		
	D3	6685		
	D4	8782		
	D5	7863		
θ = 90°	E1	10,100	9827	2.89
	E2	10,000		
	E3	9380		
	E4	9932		
	E5	9747		

**Table 4:** Poisson’s ratio testing results of wall samples with different angles of bed joints θ.

Load Angle	Ordinal No.	Observed Poisson’s Ratio	Mean Poisson’s Ratio	
		*ν_xy,_*_obs_	*ν_xy,_*_mean_	CoV (%)
θ = 0°	A1	0.16	0.156	7.40
	A2	0.17		
	A3	0.14		
	A4	0.16		
	A5	0.15		
θ = 22.5°	B1	0.16	0.182	23.76
	B2	0.15		
	B3	0.25		
	B4	0.15		
	B5	0.2		
θ = 45°	C1	0.26	0.246	14.82
	C2	0.27		
	C3	0.19		
	C4	0.23		
	C5	0.28		
θ = 67.5°	D1	0.24	0.290	12.19
	D2	0.30		
	D3	0.27		
	D4	0.31		
	D5	0.33		
θ = 90°	E1	0.24	0.220	10.66
	E2	0.19		
	E3	0.23		
	E4	0.24		
	E5	0.20		

## Data Availability

The data presented in this study are available on request from the corresponding author. The data are not publicly available due to the privacy restrictions.

## References

[1] Bajno D., Bednarz L., Matkowski Z., Raszczuk K. (2020). Monitoring of Thermal and Moisture Processes in Various Types of External Historical Walls. Materials.

[2] Liritzis I., Al-Otaibi F., Kilikoglou V., Perdikatsis V., Polychroniadou E., Drivaliari A. (2015). Mortar analysis of wall painting at Amfissa Cathedral for conservation-restoration purposes. Mediterr. Archaeol. Archaeom..

[3] Michalopoulou A., Maravelaki N.P., Stefanis N.A., Theoulakis P., Andreou S., Kilikoglou V., Karatasios I. (2020). Evaluation of nanolime dispersions for the protection of archeological clay-based building materials. Mediterr. Archaeol. Archaeom..

[4] (2013). PN-EN 1996-1-1+A1 Eurocode 6—Design of Masonry Structures—Part 1-1: General Rules for Reinforced and Unreinforced Masonry Structures.

[5] Małyszko L., Jemioło S., Bilko P., Gajewski M. (2015). FEM and Constitutive Modelling in Failure Analysis of Masonry Structures. Implementation and Examples.

[6] Małyszko L., Orłowicz R. (2000). Konstrukcje Murowe. Zarysowania i Naprawy. (Structural Masonry. Cracks and Repairs).

[7] Jemioło S., Małyszko L. (2013). MES i Modelowanie Konstytutywne w Analizie Zniszczenia Konstrukcji Murowych. Tom 1. Podstawy Teoretyczne.

[8] Abdelmoneim Elamin Mohamad A.-B., Chen Z. (2016). Experimental and Numerical Analysis of the Compressive and Shear Behavior for a New Type of Self-Insulating Concrete Masonry System. Appl. Sci..

[9] Lin K., Totoev Y.Z., Liu H., Wei C. (2015). Experimental Characteristics of Dry Stack Masonry under Compression and Shear Loading. Materials.

[10] Jasiński R., Drobiec Ł., Mazur W. (2019). Validation of Selected Non-Destructive Methods for Determining the Compressive Strength of Masonry Units Made of Autoclaved Aerated Concrete. Materials.

[11] Lourenco P.B. (2002). Computations on historic masonry structures. Prog. Struct. Eng. Mater..

[12] Beconcini M.L., Croce P., Formichi P., Landi F., Puccini B. (2021). Experimental Evaluation of Shear Behavior of Stone Masonry Wall. Materials.

[13] Malomo D., DeJong M.J. (2021). A Macro-Distinct Element Model (M-DEM) for out-of-plane analysis of unreinforced masonry structures. Eng. Struct. June.

[14] Lourenço P.B., Rots J.G., Blaauwendraad J. (1998). Continuum model for masonry: Parameter estimation and validation. J. Struct. Eng..

[15] Celano T., Argiento L.U., Ceroni F., Casapulla C. (2021). In-Plane Behaviour of Masonry Walls: Numerical Analysis and Design Formulations. Materials.

[16] Croce P., Beconcini M.L., Formichi P., Landi F., Puccini B., Zotti V. (2021). Bayesian Methodology for Probabilistic Description of Mechanical Parameters of Masonry Walls. ASCE-ASME J. Risk Uncertain. Eng. Syst. Part A Civ. Eng..

[17] Zhang S., Mohadeseh S., Mousavi T., Richart N., Molinari J.F., Beyer K. (2017). Micro-mechanical finite element modeling of diagonal compression test for historical stone masonry structure. Int. J. Solids Struct..

[18] Mojsilović N. A Discussion of Masonry Characteristics Derived from Compression Tests. Proceedings of the 10TH Canadian Masonry Symposium.

[19] Senthivel R., Sinha S.N., Madan A. Influence of bed joint orientation on the stress-strain characteristics of sand plast brick masonry under uniaxial compression and tension. Proceedings of the 12TH International Brick/Block Masonry Conference.

[20] Jasiński R. (2019). Research on the Influence of Bed Joint Reinforcement on Strength and Deformability of Masonry Shear Walls. Materials.

[21] (1989). ASTM E72 1989 Standard Tests for Conducting Strength Tests on Panels for Building Construction.

[22] Małyszko L. (2005). Modelowanie Zniszczenia w Konstrukcjach Murowych z Uwzględnieniem Anizotropii.

[23] Jasiński R. (2005). Nośność I Odkształcalność Zbrojonych Ścian Murowych Ścinanych Poziomo (Strenght and Deformability of Reinforced Clay Brick Masonry Horyzonatally Sheared).

[24] Drobiec Ł., Kubica J., Piekarczyk A. (1999). Some Remarks on Poisson’s Ratio of Unreinforced Clay Brick Masonry. Proceedings of the 77th International Scientific Conference.

[25] Capozucca R. Masonry Panels with Different Mortar Joints under Compression. Proceedings of the 13th International Brick and Block Masonry Conference.

[26] Hoffmann G., Schubert P. Compresslve strength of masonry parallel to the bed joints. Proceedings of the 10th IB2MaC.

[27] Sentler L. (1996). Tests of Swedish Masonry. Mason. Int..

[28] Drobiec Ł., Piekarczyk A., Kubica J. AAC Bocks Masonry Compressed Perpendicular and Parallel to the Bed Joints. Proceedings of the 12th International Brick/Block Masonry Conference.

[29] Bednarz Ł., Drygała I., Dulińska J., Jasieńko J. (2021). Study of Materials Behavior in a Monumental Vault Strengthened by a Carbon Net in a Mineral Matrix Subjected to Seismic Influence. Appl. Sci..

[30] Jasienko J., Raszczuk K., Frąckiewicz P., Kleszcz K., Bednarz Ł. (2021). Strengthening of masonry rings with composite materials. Herit. Sci..

[31] Page A. (1981). The biaxial compressive strength of brick masonry. ICE Proc..

[32] Dhanasekar M., Kleeman P., Page A. (1985). Biaxial Stress-strain Relations for Brick Masonry. J. Struct. Eng. ASCE J. Struct. Eng. ASCE.

[33] Dhanasekar M., Kleeman P.W., Page A.W. (1985). The failure of brick masonry under biaxial stresses. ICE Proc..

[34] Page A. (1983). The strength of brick masonry under biaxial compression-tension. Int. J. Mason. Constr..

[35] Guggisberg R., Thürlimann B. (1988). Versuche zur Festlegung der Rechenwerte von Mauerwerksfestigkeiten.

[36] Kania T., Derkach V., Nowak R. (2021). Testing Crack Resistance of Non-Load-Bearing Ceramic Walls with Door Openings. Materials.

[37] Drysdale R., Hamid A., Baker L. (1999). Masonry Structures: Behavior and Design.

[38] Radosław J. (2017). Badania i Modelowanie Murowych Ścian Usztywniających. Research and Modeling of Masonry Shear Walls.

[39] Hendry A.W., Sinha B.P., Davies S.R. (2004). Design of Masonry Structures.

[40] Ng’Andu B.M. (2006). Bracing Steel Frames with Calcium Silicate Element Walls.

[41] (2021). ASTM E519/E519M-21, Standard Test Method for Diagonal Tension (Shear) in Masonry Assemblages.

[42] Ali A.A.A., Jony H.H. (2013). Shear Wall Analysis Using Framework Method: Comparison with Shell Element Method and Column Analogy. Eng. Technol. J..

[43] Roca P. (2006). Assessment of masonry shear-walls by simple equilibrium models. Constr. Build. Mater..

[44] Roca P., Viviescas Á., Lobato M., Gomez C., Serra I. (2011). Capacity of Shear Walls by Simple Equilibrium Models. Int. J. Archit. Herit..

[45] Kok Choon V. (2006). Bracing Capacity of Partially Grounted Concrete Masonry Walls with Openings.

[46] (2015). En 772-1+a1 Methods of Test for Masonry Units. Determination of Compressive Strength.

[47] (2020). EN 1015-11 Methods of Test for Mortar for Masonry. Determination of Flexural and Compressive Strength of Hardened Mortar.

[48] (2002). EN 1052-3 Methods of Test for Masonry. Determination of Initial Shear Strength.

[49] (1998). EN 1052-1 Methods of Test for Masonry. Determination of Compressive Strength.

[50] (1988). RILEM TC 76-LUM General recommendations for methods of testing load-bearing masonry. RILEM Publ. SARL.

